# “No one talks about it, but everyone knows that it exists”: a qualitative study of nursing students’ perspectives on racism in healthcare in Norway

**DOI:** 10.1186/s12912-026-04738-1

**Published:** 2026-05-20

**Authors:** Marthe Bjørgum, Mojtaba Vaismoradi, Gøril Ursin, Rita Solbakken, Kari Ingstad, Cathrine Moe

**Affiliations:** 1 Faculty of Nursing and Health Sciences, Nord University, Bodø, 8049 Norway; 2 Faculty of Science and Health, Charles Sturt University, Orange, NSW 2800 Australia

**Keywords:** Health justice, Racism, Nursing, Nursing students, Discrimination, Healthcare, Nursing education

## Abstract

**Background:**

Despite efforts to reduce health disparities, racism is recognized as a global public health crisis. Addressing racism aligns with international healthcare policies and healthcare goals of promoting health justice. Nursing students, as future healthcare workers, are key to re-establish social patterns. This study aims to explore nursing students’ experiences of racism in healthcare and their perspectives on how such situations can be avoided.

**Methods:**

Qualitative data were collected in two phases through written reflections upon case scenarios and individual interviews of nursing students. Data were analysed through an inductive content analysis approach.

**Results:**

The data analysis led to developing one theme `there is an urgent need for ways to handle and prevent racism’ and four categories. Our results clearly reveal the presence of both overt and covert racism in healthcare, influencing professional integrity and patient rights. Racism in healthcare has a complex and multifaceted nature and an expectation that nurses respond to racist behaviour toward patients and colleagues is emphasized. However, it is difficult to identify racism and react in ‘real life’ than in a hypothetical situation. Reactions to racist behaviour depend on patient’s background and health condition. Sharing of experiences, increased knowledge of cultural sensitivity, and clear healthcare leadership are strategies that help with preventing racism in healthcare.

**Conclusions:**

This study enhances international understanding of racism within healthcare, as experienced by nursing students. It underscores that racism is not always overt or easily identifiable. Also, nursing students often feel unprepared to respond effectively in real-life situations, underscoring the need for education and training on how to address racism in practice and thereby promote health justice.

**Clinical trial number:**

Not applicable.

**Supplementary Information:**

The online version contains supplementary material available at 10.1186/s12912-026-04738-1.

## Introduction

Racism encompasses a wide array of social phenomena such as systematic inequality, institutional discrimination, internalized stereotypes and racial attitudes [1], and is central in reducing health justice [2]. For this study, racism is viewed as “…any distinction, exclusion, restriction or preference based on race, colour, descent, or national or ethnic origin” [3, para.2]. Racism harms health, consisting of adverse physical, mental, social and economic exposure. The scientific evidence in international literature is strongest for adverse effects of racism on mental health, psychological wellbeing, and related health practices (e.g., sleep disturbance, eating patterns, and the consumption of psychoactive substances, including cigarettes, alcohol and drugs) [2]. Racism is therefore a determinant of health [4]. Racialized healthcare users report moderate to high levels of race/ethnicity-based discrimination and moderate levels of socioeconomic or social class-based discrimination as key challenges in accessing healthcare [5]. Healthcare providers may fail to conduct thorough assessments, overlook patients’ symptoms, or communicate in a condescending manner [6]. Therefore, racism leads to unequal access to high-quality healthcare [7, 8] and violates the ethical commitment of healthcare professionals to provide equitable care as an act of health justice. For healthcare professionals, who prioritise avoiding harm as a core value, racism challenges their professional integrity. This may partly explain why it is not addressed openly in public discourses [9]. Despite efforts by policymakers and healthcare institutions to reduce health disparities, racism persists in subtle and systemic ways [10]. It is recognized as a global public health crisis [11].

## Background

Although nurses should be able to communicate and provide competent care to all patients, structural racism in nursing remains a global factor influencing health outcomes. Racism hampers meeting patient and family needs, raising dissatisfaction with care, while increases emotional trauma and job stress for nurses [12]. Yet there is little emphasis on open, ongoing dialogue about racism and experiences of acts of racism among nurses [13]. Therefore, addressing racism aligns with international healthcare goals and nursing policies, emphasizing the need for cultural adaptation in antiracism efforts [14].

Racism is a socially constructed system developed and reinforced over time [15]. Nursing students as future healthcare workers are key to re-establish social patterns. Systemic racism embedded in curricula and institutional practices may perpetuate bias and reinforce inequities, limiting students’ ability to fully engage and succeed academically [16]. The cultural competence of future healthcare providers should therefore be improved by addressing ethnocentrism, challenging entrenched biases, and acknowledging the scope of systemic racism to help mitigate health disparities [17]. To build cultural competence for students, nurse educators should amplify discussions on racism and encourage race-related dialogue in the classroom to address the impact of structural racism on health equity [18]. Both nurse educators and nursing students might also be familiar with racism as they experience direct and indirect racism in both clinical settings and educational environments [19, 20]. In clinical settings nursing students report racism from patients and nurses, including microaggressions, verbal assaults and discriminatory treatment. Issues reported include patients refusing care, questioning clinical competencies and lack of support from staff [19, 21, 22]. Racism is also experienced in classrooms, where students have faced discriminatory attitudes from peers and educators, including stereotyping and highlighting perceived limitations of certain ethnic groups [19, 23]. Being exposed for racism impacts mental health, overall well-being, confidence, learning opportunities and professional development for students [19, 21, 22, 24, 25, 26]. Racism affects students’ sense of belonging and learning opportunities, which undermines their educational experience and clinical readiness [20]. It can perpetuate bias and reinforce inequities, limiting students’ ability to fully engage and succeed academically [16]. It can also have clear health implications including cumulative psychological stress, emotional exhaustion, and physiological harm among students [26], leading to hindered professional development trajectories [25].

Exploring racism in nursing education settings has been conducted in geographical contexts such as US [22, 27, 28], Canada [29], UK [19, 21], and Sweden [24]. This study is conducted in Norway. The Norwegian healthcare system is public funded and based on the principles of equality and fair distribution of healthcare services. All healthcare professionals are obligated by legislation and ethical guidelines to provide healthcare protecting the inherent dignity of every individual, regardless of national origin, ancestry, skin colour, or language [30, 31]. Norway has an indigenous population, the Sàmi people. Also, the Norwegian society has been through a demographic shift with an increasing migrant population. In 2025, the population of Norway included 1 million individuals with a migration background, accounting for around 21% of the total population. This figure comprises 17,3% immigrants and 4,1% descendants of immigrants [32]. The Norwegian demographic shift and indigenous population underscore the increasing multicultural composition of the Norwegian society and its influence on various sectors, including healthcare. For instance, municipal healthcare services have increased the number of employees from immigrant backgrounds from 15.7% to 18.1% between 2016 and 2020 [33]. As of 2017, immigrants working in these services represented more than 160 different countries, with the largest groups coming from the Philippines, followed by Poland, Eritrea, Somalia, Sweden, and Thailand [34]. However, there are significant regional variations in the employment of immigrants within healthcare services. The highest concentration is in the capital, Oslo, where over 40% of full-time equivalents are performed by individuals with immigrant backgrounds [33]. There is no such statistics for the indigenous population.

Despite global calls for equity in healthcare and professional obligations to provide culturally competent care, racism remains a persistent barrier within nursing, adversely affecting both patient outcomes and nurse well-being [12]. Previous studies have highlighted the negative influence of racism on nurses and patients, and the need for culturally competent and responsive care [12]. Recurring patterns such as stereotyping, dismissal of concerns, and inequitable treatment of patients from minority backgrounds, all of which contribute to compromised care quality and patient dissatisfaction. Nurses from minority backgrounds also experience marginalisation, including being discredited or unheard within clinical teams, which may further undermine teamwork and patient safety [35].

Interventions aimed at enhancing nurses’ cultural competence can reduce inequalities, improve communication, and lead to better patient-related outcomes [36, 37]. Cultural competence is increasingly conceptualized as a dynamic process involving awareness, knowledge, skills, and ongoing engagement with diverse populations, enabling nurses to tailor care to patients’ cultural contexts and needs [38].

As most existing research on racism in nursing originates from the United States [7], there is a notable lack of empirical studies from countries with differently organized healthcare and nursing education systems, such as Norway, where welfare-state structures and demographic changes shape both healthcare delivery and professional training in distinct ways. There is also limited research explicitly focusing on how racism is experienced by nursing students, and their perspectives as future healthcare workers on how racism can be avoided. This gap is particularly relevant in Norway, where demographic shifts have led to a rapidly growing multicultural population and a more ethnically diverse healthcare workforce. Given the emerging literature calling for open dialogue, cultural competence, and anti-racist pedagogy in nursing, research is required to understand how racism is conceptualized, experienced, and addressed within nursing education. In addition, addressing racism underscores the need for research on racism aiming to reshape the discourse on health disparities and inequalities [39]. Accordingly, this study aims to explore nursing students’ experiences of racism in healthcare and their perspectives on how such situations can be avoided.

## Methods

We used a qualitative exploratory design consisting of two phases of data collection including case scenarios with open-ended questions (phase 1), and individual semi-structured interviews (phase 2). The two phases of data collection were independent but complementary. Findings from Phase 1 informed the development of additional questions, which were then incorporated into the data collection for Phase 2.

The qualitative exploratory design helped gain an in-depth understanding of participants’ perceptions and experiences, particularly in relation to complex and sensitive issues such as racism in healthcare. The use of case scenarios in phase 1 provides a structured yet flexible way to elicit participants’ reflections on realistic situations, helping to uncover implicit attitudes, decision-making processes, and context-specific responses that may not emerge through direct questioning [40, 41]. In phase 2, individual semi-structured interviews were employed to further explore and deepen the insights generated from the case scenarios. Semi-structured interviews are widely recognized for their ability to balance consistency across participants with the flexibility to probe emerging themes, clarify meanings, and capture rich, nuanced data [42].

### Study setting and recruitment

The study was conducted in a university setting in Norway. All authors are employed at the same university where the research took place. Participants were master’s and bachelor’s degree nursing students. For reflection on case scenarios, participants were recruited from a master’s-level research methods course during the academic years 2021–2022. The course leader (MV) invited students to participate voluntarily. Students received both oral and written information of the study. Participation in the study was not a mandatory component of the master’s course. For interviews, members of the research team (MB and CM) visited master’s and third-year bachelor’s classes to inform students about the study and invite them to participate. All students were orally invited to participate, with no other inclusion or exclusion criteria. Those students who chose to take part in a research interview received a gift card valued at 400 NOK for use at a local shopping centre. Students willing to participate in research interview contacted the first author by phone or e-mail to coordinate the time and location of the interview. They also received written information about the study. Altogether 16 students participated in either case scenario reflection or interview.

### Data collection

Since racism can be uncomfortable to discuss even among nursing students [28], data collection consisted of two phases: first, case scenarios with open-ended questions (phase 1), followed by individual semi-structured interviews (phase 2). Case scenarios with questions are an indirect method of data collection that invites participants to reflect upon hypothetical situations [43]. Traditionally centred on individual patient care, the use of case scenarios can be a powerful tool for understanding social determinants of health, including racism and its negative health impacts. It equips students to analyse case studies through this lens, fostering skills to address the harmful effects of systemic racism and discrimination in clinical practice [44]. Research using case scenario has been shown helpful to reveal common patterns in how discrimination impacts health and well-being [45].

The research team developed two case scenarios illustrating racism in nurse-patient relationships based on findings from the international literature. One case scenario indicated the presence of discrimination in the nurse behaviour towards the patient, and another one described racist behaviour applied by the patient towards the nurse (See Appendix A). Students were asked to describe their thoughts about the scenarios and to reflect on their own experiences with similar situations. They were also invited to suggest strategies for preventing such incidents in nursing care. 10 master students participated and reflected on the case scenarios. Their responses were handwritten and anonymously submitted to the course leader (MV).

Based on analysis of the case scenarios and identification of key aspects of racism in nurse-patient relationships from the students’ perspectives, the research team developed an interview guide for conducting complementary individual interviews. The questions in the guide were designed to encourage students to provide rich descriptions of their experiences of racism in care, drawing on the findings of case scenarios identified. They also sought to elicit suggestions for preventing discrimination and racism. The interview guide (See Appendix B) included open-ended questions and conversational topics. However, the order of questions was not followed strictly, and several probing questions prompted follow-up inquiries, following the Kvale and Brinkmann’s [46] approach to qualitative interviews.

**Fig. 1:**
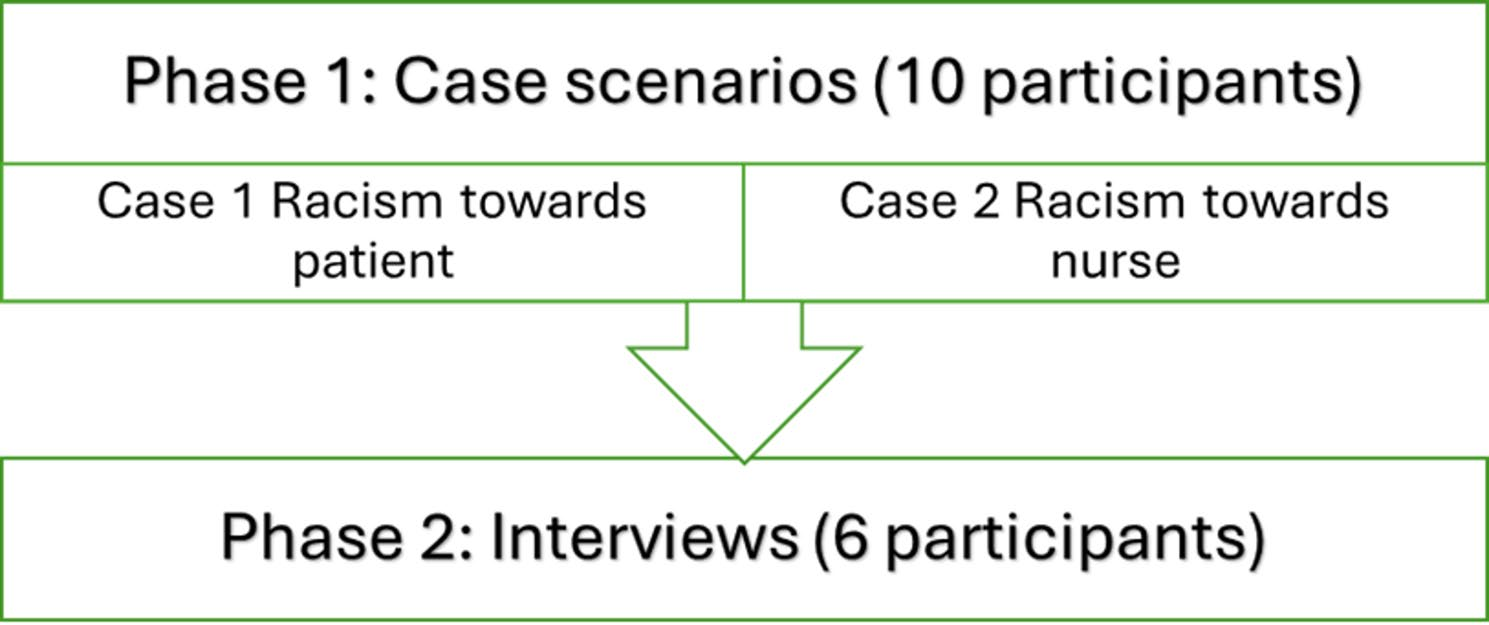
An overview of the two phases of data collection

Five face- to- face individual semi-structured interview sessions were performed in a quiet place by the first author (MB) who is qualified in qualitative research methods, along with one online interview session via Microsoft Teams. The interviews were audio recorded and lasted between 45 and 60 min. Audio recordings were made using the Nettskjema application. All interviews were transcribed verbatim using Whisper AI, an automatic speech recognition model integrated into Nettskjema. Transcripts were manually reviewed and corrected by the first author. An overview of the two phases in data collection has been depicted in Fig. 1.

### Data analysis

Qualitative content analysis was used to guide the analysis of both the written reflections on case scenarios and the transcribed interview data [47–49]. The data analysis involved several steps, beginning with the descriptions from the case scenarios. The students’ texts were read multiple times and manually coded inductively by author (MV)*.* The codes were grouped into subcategories and categories based on our interpretations of their meaning. An analytic narrative to interpret the results was composed. For the transcribed interview data, the first author (MB) read the transcripts several times and coded the data inductively relating to the aim of the study. Categories and subcategories were derived from the codes and merged with the categories from the first analytic phase. The authors (MB, MV and CM) refined the analytic text presented in findings. Table 1 shows two examples of the data analysis conducted on the data. Data collection and analysis were conducted in Norwegian. Quotes were translated to English in the last analytic and writing phase.

**Table 1:** Examples of content analysis of the interview data

Transcribed text	Code	Subcategory	Category
And when it is not so visible, it also becomes more difficult to address	Not being visible; Difficulty to address the issue	Visibility of racism	Both covert and overt racism exist in healthcare
You understand that they are avoiding you.	Being avoided		
And then I think it’s just easier to ignore it, than to try to understand, if you don’t understand already	Easier to ignore; Not understanding the incident	Coping mechanisms	Dealing with racism in healthcare
I get such a rush in my stomach, and then I need to remove myself from the situation to protect myself	Need to avoid the situation		

### Rigour, reflections on methods and reflexivity

To ensure rigour, and enhance transparency and accountability, the study was guided by established qualitative research standards, including the use of COREQ (consolidated criteria for reporting qualitative research) [50 Tong] (See Appendix C). Methodological rigour was sought by being transparent in the recruitment process, data collection and following the analytical steps outlined by Graneheim and Lundman [48], and Lindgren, Lundman and Graneheim [49]. The same analytical steps and reflexivity were maintained throughout both phases to ensure rigor. For the case scenarios, the indirect method of data collection facilitated the exploration of this phenomenon based on the students’ perspectives. Due to the sensitivity of the topic, the students were not asked directly but reflected on the scenarios by placing themselves in the described situations. Also, the anonymous format allowed them to respond more honestly and take time to provide deeper and more thoughtful answers. We engaged multiple students simultaneously and captured a diverse range of responses. For the interviews, the first author was coordinator for the third year of the bachelor programme and presented some lectures prior to the interviews to the students. Still, she did not have a close relationship with any of the students and found physical interviews to be suitable to be able to provide care to the participants. She found it easier to discover non-verbal signals and be watchful concerning participants glance and small breaks. Although all authors are employed at the university, none had a close relationship with any of the participants. The researchers acknowledged that their preconceptions significantly influenced the research process. Therefore, they engaged in continuous reflexivity, critically examining their assumptions and documenting their perspectives to enhance the transparency and credibility of the study. The research team consisted of six members—one male and five females—with one author having a non-Norwegian background. All authors are nurses, having various specialisations and clinical experiences. Ongoing discussions within the group throughout the research phases enhanced reflexivity and helped balance the influence of each researcher’s preconceptions on the interpretation of the findings.

## Findings

### Characteristics of participants

A total of 16 nursing students participated, all were female and had clinical experiences as nurse or student. The master’s degree students combined their nursing work with their coursework, while all bachelor’s students had completed 36 weeks of clinical placement as part of their education. Additionally, some bachelor’s students already held part-time jobs as nurse assistants in clinical practice. Therefore, experiences shared in the interviews and case scenario reflections came from various job settings. Participants were university students from different regions of the country. Table 2 shows an overview of the study participants.

**Table 2:** The demographic characteristics of the study participants in each phase of data collection

Participant ID	Age, year	Educational level	Workplace	Years of nursing experience	Data collection
1	38	Master’s degree	Municipal Home care	9	Case scenarios
2	34	Master’s degree	Hospital care	8.5	Case scenarios
3	44	Master’s degree	Municipal Nursing home	5.5	Case scenarios
4	28	Master’s degree	Case management, allocation office	10	Case scenarios
5	45	Master’s degree	Municipal Home care	6	Case scenarios
6	45	Master’s degree	Municipal Home care	6	Case scenarios
7	39	Master’s degree	Municipal Nursing home	10	Case scenarios
8	37	Master’s degree	Municipal Home care	17	Case scenarios
9	37	Master’s degree	Municipal Home care	17	Case scenarios
10	27	Master’s degree	Municipal Home care	10	Case scenarios
11	22	Bachelor’s degree	n/a	n/a	Interview
12	26	Master’s degree	Hospital care	4	Interview
13	28	Master’s degree	Hospital care	5	Interview
14	24	Bachelor’s degree	n/a	n/a	Interview
15	21	Bachelor’s degree	n/a	n/a	Interview
16	32	Bachelor’s degree	n/a	n/a	Interview

### Experiences of racism in healthcare: urgent need for ways to handle and prevent racism

The inductive analysis from the data collected using case scenarios and interviews collectively resulted in developing one theme and four categories as presented in Table 3. The theme “Urgent need for ways to handle and prevent racism” illustrates the critical need to address and respond to racism in healthcare. Racism negatively affects the health of both patients and nurses, and addressing and preventing racism is a prerequisite for health justice. The categories presented below further explain and refine the theme. Within the findings, it is specified which results belong to each phase of data collection; however, to ensure a coherent and complementary presentation, they are organized under the same categories.

**Table 3:** Overview of the data analysis results

Theme			
Urgent need for ways to handle and prevent racism			
**Categories**			
Both covert and overt racism exist in health care	Racism hampers professional integrity and patient rights	Dealing with racism in healthcare	Preventive measures against racism in healthcare

### Both covert and overt racism exist in health care

All interviewed participants had personal experience with racism in healthcare, either as a witness of colleagues having racist behaviour towards patients or other colleagues or being exposed to racism themselves. “No one talks about it, but everyone knows that it exists.” (Participant 15). From case scenarios, almost all participants wrote in their notes that they had experienced episodes of racism as described in the case scenarios. Participant 4 wrote: “I had a fellow student who was not allowed entering into a patient home in homecare due to the skin colour.”

Descriptions of situations with racism, involved experiences of “covert racism” such as avoidance behaviour. From the interviews, it was further identified that it could involve a patient delaying care activities until a preferred nurse is on duty, or nurses avoiding visits to patients from different ethnic backgrounds. “Racism shows more indirectly. You understand that they are avoiding you. For white students it has not been a problem at all. But when I arrive, they are not interested in my care.” (Participant 15).

Participants described how such a behaviour often was justified by language problems. Also, several participants brought up humour to conceal racism. They described covert racism through humour as being most common among younger people. When racist statements came in the form of humorous formulations, it was difficult to react to them. It could also be hard to distinguish between racism and humour, and to know whether a person was ironic or not. Some participants in the interviews expressed that it was hard to react to humours situations because they did not want to be perceived as humourless or strict.

You probably have a higher threshold for speaking up when something is being laughed about around the table [.]. Racism comes in a different form [through humour], which is difficult to react to. It may appear more innocent, but it is not. But… you don’t want to be perceived as humourless. (Participant 14)

Within both case scenarios and interviews, participants described overt racism mainly from two groups: older people and individuals with cognitive impairments, particularly those with severe mental illness and dementia. People with dementia were highlighted as a group where participants had experienced the most pronounced racism. “I have often experienced that elderly patients with dementia are most outspoken. Like commenting that ‘I want a Norwegian nurse, I don’t want a black person, I don’t want a burned person’.” (Participant 16).

Participants who had experienced racism themselves from older patients and patients having cognitive impairment, explained that it was easier to deal with emotionally than racism from cognitively healthy people. Participants mentioned that older and cognitive impaired people could be excused because they had grown up in a completely different time where racist statements were more accepted, or that the cognitive impairment was the cause of statements. “We had a patient who was delirious, who was very rude to one of my colleagues. The patient was very ashamed of it afterwards. He said a lot of rude things during a period when he was not cognitively in place. But the patient, my colleague and I, we had a chat afterwards.” (Participant 13).

### Racism hampers professional integrity and patient rights

Participants considered nurse’s racist behaviour from case scenario as violating the patient’s right. The nurse’s behaviour depicted in the case scenario was attributed to her racist attitude and being impatient. Further in the interviews, it was stated: “I believe the patient is unsure and unsafe. Has she [the patient] understood what the nurse informed about? Is this the first time she was hospitalized or maybe the first time she got her blood pressure measured?” (Participant 10). Although patients should know about the care process, participants thought there was no need for the patient to be informed of the nurse’s workload. Participants were unsure if a white person would the treated the same way. Participants expressed a need for nurses to create a feeling of safety among all patients in health services and to ensure the nursing integrity in healthcare.

For the case scenario involving racist behaviour by a patient, the patient’s behaviour was considered unacceptable and irritating to be judged. The professional integrity of the nurse was considered offended. Still, participants agreed that the nurse was obliged to uphold the patient rights for care and the incident should not hinder nurses to provide care to the patient. According to participants, in the interviews, all patients should be respected: “We must respect the patient and give time to adapt to such situations” (Participant 3). Still, participants agreed that patients cannot choose nurse based on the nurse’s background. Even though this agreement, the interviews revealed several accounts of patients avoiding care from nurses and nursing students: “I have experienced some patients who do not want my help because of the way I look like.” (Participant 15).

### Dealing with racism in healthcare

From case scenarios, participants who had personally experienced racism reported that they had felt unprepared for such incidents. Therefore, they had no strategies themselves for handling them. They described various experiences of how it was handled by others. One form of reaction from a supervisor, colleague or leader was to trivialise the incidents. Immediate reactions were also described. One participant described episodes where she herself had been usure how to interpret incidents, whereupon a colleague referred to them as racially motivated. Based on the interview findings, none of the participants had experienced any form of follow-up after being exposed to racism. They described a need to withdraw from the situations to protect themselves. Participants shared they had private networks who helped them to process the burden of being exposed to racism.

What I’ve learned about myself is that when I react to such situations, it’s like I get a real shock and then I must remove myself from the situation because I don`t automatically get angry. I get such a rush in my stomach, and then I need to remove myself from the situation to protect myself. (Participant 11)

In reflection upon case scenarios, participants acknowledged how hard it must be being exposed to racism. Relating to participants, a response to racist utterances from patients could be to ignore insults from patients or stop the care for a moment to give both the patient and the nurse some time. For the exposed nurse, participants in the interviews expressed it to be essential to report racism to the close leader or ask for support from colleagues. “I have experienced the same myself. There was a patient that did not like me because of my foreign origin. I reported it to my leader. To stop racism everyone must say that all nurses working in the unit is equally worth and that we do not accept racism.” (Participant 1).

Participants expressed a desire to stand up against racism depicted in the case scenarios, but several of them experienced that it could be hard. Especially in the role of student or assistant where one feels hierarchically inferior, they described it in the interviews difficulties in speaking up.

Well, you can talk to your nurse supervisor. Because as a student, it’s a bit scary, especially if you’re in your first hospital clinical placement. Confronting directly the patient on their racist behaviour can be scary. Having support from someone who is more experienced and who may be able to approach the patient in a different way is important. (Participant 15)

One participant described a deliberate strategy of promoting positive experiences of working with both colleagues and patients from other countries and cultures.

I stand up for my colleagues, those with different ethnic backgrounds. Simply because I don’t want to contribute to it getting worse, and for the gap between us to become bigger. I don’t want to smile and follow along when others are racists. (Participant 14)

Within participants’ accounts, there was broad agreement that healthcare service environment needed culture change in the form of practice reflection and discussions on racism incidents.

### Preventive measures against racism in healthcare

All participants in both the interviews and case scenarios emphasized that focusing on racism and fostering openness around the topic are essential for preventing it. They described that they had learned little or nothing about racism during education or in clinical practice. All participants expressed a desire to engage with it and a need for increased knowledge about it.

Suggestions for how to put racism on the agenda was to encourage sharing experiences of racism. In that way everyone can be prepared when they find themselves in such incidents. Situations can come as a surprise, leading to nurses not always reacting in the way they want. Sharing experiences also provides more confidence in putting them into words. In the interviews, one of the participants raised a concern about too much publicity on racism in healthcare. She feared it could scare people away from wanting to work as a nurse. She would rather speak about the benefits of multicultural colleagues: “There are many nice stories, and we need to talk to them directly. I would say that some of the best people I’ve worked with were people from other countries, and I talk about these positive experiences.” (Participant 16).

According to participants, nursing educators should take greater responsibility for focusing on racism in healthcare. Participants expressed that they need guidance of how to prevent racism and how to deal with racist behaviour. All participants expressed a need for clearer attitudes against racism within healthcare. Particularly leaders should be visible, clear and held accountable. “I think it’s about making students and nurses prepared to dare to ask questions.” (Participant 13).

Participants suggested courses in cultural understanding and cultural sensitivity. In addition, the need for ongoing work with a culture of openness was highlighted so nurses can be comfortable of talking about racism. More and better use of interpreting services in healthcare was highlighted as a concrete and important factor for preventing racism. Also, recruitment of nurses and other healthcare professionals from various ethnic backgrounds was proposed as a measure to prevent racism: “It is important that we have work colleagues who come from different cultures.” (Participant 12).

Participants emphasized that prevention of racism should be planned and systematised and included in patient safety campaigns and quality improvement initiatives.

When we graduate, we have an idea of how we should be and behave. But we are shaped by the culture at the workplace we enter. It doesn’t matter how much we fight if they don’t want changes. And then eventually we are shaped by that culture. I think it needs to be changes in workplaces. It could either be courses, or having a thematic day, to focus on racism out there. I think it’s important to change cultures. (Participant 16)

## Discussion

This study explored nursing students’ experiences of racism in healthcare and their perspectives on how such situations could be avoided in a Norwegian educational context. Our results underscore an expectation that nurses take responsibility for addressing and responding to racist behaviours, whether these are directed at patients or their colleagues. However, it is more difficult to identify racism and react in ‘real life’ than in a hypothetical situation. There is no clear line for when an episode is considered racist, and racism can be covered by avoidant behaviour and humour. Reactions to racist behaviour are influenced by patient’s background and health condition. Utterances from patients experiencing severe mental illness or cognitive impairments are easier to deal with emotionally, also reported by Caffrey et al., [21]. According to our study findings, nursing students recognize the negative impact of racism on patient rights and the healthcare environment, but they report a significant gap in knowledge, preparation, and support to address racist incidents when they occur. Sharing experiences, enhancing knowledge of cultural sensitivity, and proactive healthcare leadership are strategies for preventing racism in healthcare. Even if most existing research on racism in nursing originates from the United States [7], exploring racism in nursing education settings has been conducted in other geographical contexts [19, 21, 24, 28, 29]. Accordingly, the presence of racist behaviours and the complex and multifaceted nature of racism in healthcare have been identified offering insights into how nursing students perceive, experience, and manage racism within nursing interactions in healthcare services. In this respect, the research findings have been discussed in connection to the international knowledge of the topic under three dimensions of the complex and multifaceted nature of racism in healthcare, the need for innovative educational methods in nursing, and the important role of leadership and systemic anchoring.

### The complex and multifaceted nature of racism in healthcare

Racism is a highly sensitive and challenging issue that often evokes anxiety, shame, blame, anger and guilt among nurses, nursing students, and nurse educators [13]. Racism is characterised as a global public health crisis [11] and it can result in significant health injustice. When developing our case scenarios, we deliberately made the racist behaviour explicit, requiring students to respond with a clear and unequivocal stance against racism. Based on these scenarios, responding to such incidents appeared relatively straightforward, and participants offered clear and structured descriptions of how racism should be addressed. Our interviews, however, provided insight into the complexity and multifaceted nature of racist behaviour, which is not as clear-cut as it may appear from the literature. Racism was described as overt, primarily from older adults or individuals with cognitive impairments. Racism was also sometimes covert, making it subtle and difficult to respond to. This may help explain why racism in healthcare remains under-investigated [13].

Racism does not base only in colours of black and white, but in race, religion, descent or national or ethnic origin [3]. It leads to differences on how people are treated based on subtle differences. A collective silence can maintain racist behaviour, having unintended consequences impacting the nursing profession [51]. As racism is a determinant of health [4] and central in reducing health justice [2], it is essential for healthcare services to develop interventions specifically addressing racism, and for nurse educators to address such sensitive issues as pointed out by Miller and Nambiar-Greenwood [19].

### The need for innovative educational methods in nursing

A main concern raised by participants within both case-scenarios and interviews was the lack of knowledge among students regarding racism. Also, many participants reported feeling unprepared and having limited experience in discussing how to manage such situations. This finding underscores the need for nurse educators to address the sensitive issue of racism within nursing education. Since racism is covert, complex, and multifaceted, combined with the fact that nursing students often feel unprepared when encountering it, requires pedagogical strategies to enhance students’ awareness, critical reflection, and confidence in recognizing and responding to such situations in clinical practice. Therefore, pedagogical opportunities should be provided for students to encounter realistic situations to respond in real-life circumstances. For instance, flipped classroom can create safe space for discussing racism [19]. Also, simulation-based teaching offers an approach to strengthen students’ ability to identify and address everyday challenges. It uses realistic scenarios to mimic clinical situations, allowing students to practice and refine skills in a safe, controlled environment. Although its learning effects remain uncertain [52], simulations can range from simple role-play to complex, high-fidelity experiences using advanced technology [52]. Case scenarios involving racial bias and discrimination can be integrated into simulation-based teaching to help students recognize and respond to racism without risking harm to real patients [53]. Developing empathy by placing oneself in the position of patients or colleagues who experience racism can help cultivate a compassionate and culturally competent nursing workforce. Students who engage in such learning activities are more likely to recognize and challenge their own biases, contributing to improved patient care and outcomes [54]. Addressing subtle microaggressions and the ‘bystander effect’ where individuals may be less likely to intervene can further prepare students to respond effectively in practice [55]. Additionally, it is important to create safe spaces for students from minority backgrounds in ways that do not reinforce prejudice or stereotypes [56].

### The important role of leadership and systemic anchoring

Educating nursing students and building cultural competence are important, but they are not sufficient on their own. Our findings show the important role of a clear healthcare leadership against racism. Healthcare institutions should adopt a multi-level, long-term approach to anti-racism that includes systemic changes, and ongoing evaluation of interventions to effectively address and dismantle racism in healthcare settings [43].

Leadership plays a crucial role in shaping workplace culture and addressing systemic challenges such as racism in healthcare settings. Nurse leaders are uniquely positioned to influence both structural and interpersonal dynamics by setting clear expectations, modelling inclusive behaviours, and promoting accountability across teams [14]. Also, nurse leaders should educate new nurses about racism [24]. Therefore, fostering an anti-racist culture requires leaders to move beyond passive endorsement and actively engage in identifying, confronting, and dismantling racism. Such efforts become particularly complex in dementia care, as highlighted by our participants, where patients may exhibit racist behaviours because of cognitive impairments. It is essential that nurse leaders acknowledge these difficulties and provide proactive support through guidelines, space for ethical reflection, and facilitate structured, team-based strategies. To meaningfully address and mitigate racism in healthcare, nurse leaders should be engaged and equipped with actionable plans to confront both interpersonal and structural racism [57]. They should also move beyond superficial competence and embrace racial humility. This is an ongoing and reflective practice that requires courage, self-assessment and willingness to disrupt historical inequities, as requested by our study participants. This includes setting clear expectations for anti-racism [58]. Effective anti-racism action requires leaders to dedicate resources, support and funding of ongoing, mandatory and tailored education for staff. This education needs to address both implicit and explicit biases [56].

Our study highlights situations where doubt and uncertainty about responsibility and action options were prominent. All nurses are expected to demonstrate leadership in daily practice; consequently, leadership skills are widely recognized as essential for newly graduated nurses [27]. Leadership training increases new nurses’ confidence and ability to act as change agents [59]. This indicates a connection between strengthening the focus on leadership skills in education and combating racism in healthcare.

### Strengths and limitations of the work

This study employs a qualitative exploratory design with two complementary phases, enhancing the depth and richness of the data. The use of case scenarios allowed participants to engage thoughtfully with realistic and complex situations, and the follow-up individual interviews provided an opportunity to explore participants’ perspectives in greater detail. The combination of these methods supports a comprehensive understanding of the topic and advances the role of qualitative methods in promoting health justice. Additionally, involving students with clinical experience ensured that the insights were grounded in practical knowledge.

Despite these strengths, the sample consisted solely of female nursing students from one university, which may limit the transferability of the findings to broader or more diverse populations, including male nursing students. A limitation of the study is the relatively small number of participants. However, data saturation was reached after six interviews, suggesting sufficient depth and consistency in the findings. The sensitive nature of the topic might have influenced participants’ willingness to share openly, leading to social desirability bias. Furthermore, the case scenarios rely on hypothetical scenarios, which may not fully capture the complexity of real-life situations. By not inviting the same participants who responded to the case scenarios for follow-up interviews, we were unable to explore their perspectives in greater depth. This may be viewed as a limitation of the study, as it limited the opportunity to obtain a more nuanced and in-depth understanding of their responses.

### Recommendations for further research

There is still a need for further research to explore ways to promote health justice through preventing and effectively reacting to racism in healthcare, with a particular focus on integrating these approaches into nursing education. Future research should inform curriculum development, training programs, and practical interventions to better prepare nurses to respond to racist behaviours and promote equity in clinical practice.

### Implications for policy and practice

At the policy level, our results underscore the need for national and institutional strategies to address racism within healthcare and nursing education. Recognising racism as a social determinant of health is essential for developing equitable workplace policies. Furthermore, at practice level, training in cultural competence should be integrated into nursing education curricula and within healthcare services. Creating inclusive and supportive work environments are crucial for protecting both patients and nurses, improving health outcomes and ensuring health justice.

## Conclusion

This study enhances understanding of the complex and multifaceted nature of racism within healthcare, as experienced by nursing students. It underscores that racism is not always overt or easily identifiable. Nursing students often feel unprepared to respond effectively in real-life situations. Racism is a determinant of health and there is a need for systemic and educational interventions for prevent and handle racism in healthcare. The findings of this study can inform the development of culturally adapted strategies that promote health justice, inclusion, equity, and preparedness among future nurses, thereby contributing to the prevention of discrimination and racism within nurse-patient relationships and supporting equitable health outcomes for all.

## Supplementary Information

Below is the link to the electronic supplementary material.

Supplementary Material 1

## Data Availability

The datasets generated and analysed during the current study are not publicly available due to participant confidentiality but is available from the corresponding author on reasonable request.

## Abbreviations

COREQ: Consolidated criteria for reporting qualitative research
SIKT: Norwegian Agency for Shared Services in Education and Research
