# Supplementary material for: “No one talks about it, but everyone knows that it exists”: a qualitative study of nursing students’ perspectives on racism in healthcare in Norway
Source: BMC Nurs. 2026 May 20;25:621. doi: 10.1186/s12912-026-04738-1 (PMC13366750; doi:10.1186/s12912-026-04738-1)
Supplement: Supplementary file 1 — Supplementary Material 1 [file 12912_2026_4738_MOESM1_ESM.docx]

**Appendix A. Case scenarios**

Case scenario 1. Racist behavior of the nurse towards the patient

The nurse enters the room of an overweight black woman and informs her that she is going to measure her blood pressure. The nurse asks the patient to get ready for the procedure and instructs the patient to sit in the correct position and undress her right arm. The patient does so slowly and does not fully uncover the entire arm. The nurse becomes agitated and says: 'I don't have this problem with regular patients, you need to cooperate and do it quickly because I'm very busy today'.

Open-ended questions to be answered:

1. Describe your initial thoughts about what is happening in the scenario.
2. Which parts of the scenario caught your attention or were surprising to you, and why?
3. Have you experienced a similar situation yourself, or have you observed or heard of a similar situation? Describe it.
4. What are your suggestions for preventing such an incident in clinical practice?

Background information:

Age: Gender: Workplace:

Number of years of experience in clinical practice:

Case scenario 2. Racist behavior from the patient towards the nurse

The nurse has an immigrant background and is recently graduated from university. She is employed as a nurse responsible for patient care and is about to measure the blood pressure of a male patient and record it in his medical records. She enters the patient's room and informs him of what she is going to do. He responds that he prefers a nurse with the same skin color to perform the procedure and then asks the nurse to leave the room.

Open-ended questions to be answered:

1. Describe your initial thoughts about what is happening in the scenario.
2. What parts of the scenario caught your attention or were surprising to you, and why?
3. How should the nurse react to this incident and why?
4. Have you experienced a similar situation yourself, or have you observed or heard of a similar situation? Describe it.
5. What are your suggestions for preventing such an incident in clinical practice?

Background information

Age: Gender: Workplace:

Number of years of experience in clinical practice:

**Appendix B. Interview guide**

**Racism in healthcare**

1. Do you have any experience of caring for patients with other cultural/ethnic backgrounds?
2. Have you previously heard of racism in healthcare? How would you describe racism from your perspective?
3. Do you have any experiences of racism in healthcare services, either against the patient or against the nurse (or other health personnel)?

- Is this experience personal or is it something you know that others have experienced?
- Have you experienced racism between nurses (e.g. in the distribution of tasks).

1. Can you tell us about/describe this incident?

– Direct or indirect racism?

1. What do you think this is about?
2. What options do nurses have in such a situation?
3. How can such incidents be avoided?
4. What type of knowledge or expertise would be desirable/necessary to prevent racism in healthcare services?

- At what level will it be natural to increase knowledge for it to contribute to a positive change? In education/in the workplace?

1. What can be done at workplaces to contribute to a safe environment for patients and nurses?
2. Do you have anything else to add, or is there something I forgot to ask you about?
